# Biological importance of OCT transcription factors in reprogramming and development

**DOI:** 10.1038/s12276-021-00637-4

**Published:** 2021-06-11

**Authors:** Kee-Pyo Kim, Dong Wook Han, Johnny Kim, Hans R. Schöler

**Affiliations:** 1 Department of Cell and Developmental Biology, Max Planck Institute for Molecular Biomedicine, Röntgenstrasse 20, Münster, 48149 Germany; 2 Department of Life Sciences, College of Medicine, The Catholic University of Korea, 222 Banpo-daero Seocho-gu, Seoul, 06591 Republic of Korea; 3 Guangdong Provincial Key Laboratory of Large Animal models for Biomedicine, Wuyi University, Jiangmen, 529020 China; 4 Department of Cardiac Development and Remodeling, Max-Planck-Institute for Heart and Lung Research, Bad Nauheim, 61231 Germany; 5 Medical Faculty, University of Münster, Münster, Germany

**Keywords:** Reprogramming, Induced pluripotent stem cells

## Abstract

Ectopic expression of Oct4, Sox2, Klf4 and c-Myc can reprogram somatic cells into induced pluripotent stem cells (iPSCs). Attempts to identify genes or chemicals that can functionally replace each of these four reprogramming factors have revealed that exogenous Oct4 is not necessary for reprogramming under certain conditions or in the presence of alternative factors that can regulate endogenous Oct4 expression. For example, polycistronic expression of Sox2, Klf4 and c-Myc can elicit reprogramming by activating endogenous Oct4 expression indirectly. Experiments in which the reprogramming competence of all other Oct family members tested and also in different species have led to the decisive conclusion that Oct proteins display different reprogramming competences and species-dependent reprogramming activity despite their profound sequence conservation. We discuss the roles of the structural components of Oct proteins in reprogramming and how donor cell epigenomes endow Oct proteins with different reprogramming competences.

## Stem cells: Mechanisms for reprogramming cells

Cells can be reprogrammed into induced pluripotent stem cells (iPSCs), embryonic-like stem cells that can turn into any cell type and have extensive potential medical uses, without adding the transcription factor OCT4. Although other nearly identical OCT family members had been tried, only OCT4 could induce reprogramming and was previously thought to be indispensable. However, it now appears that the reprogramming can be induced by multiple pathways, as detailed in a review by Hans Schöler, Max Planck Institute for Biomolecular Medicine, Münster, and Johnny Kim, Max Planck Institute for Heart and Lung Research, Bad Nauheim, in Germany. They report that any factors that trigger cells to activate endogeous OCT4 can produce iPSCs without exogeously admistration of OCT4. The mechanisms for producing iPSCs can differ between species. These results illuminate the complex mechanisms of reprogramming.

## Introduction

The OCT protein family comprises eight transcription factors that bind to specific target sequences to regulate gene expression^1–3^. These factors play important roles in the maintenance of cellular identity in different tissues and in mediating cell fate decisions throughout embryonic development^4–6^. Because Oct proteins regulate the expression of hundreds of target genes that determine and maintain cellular identity, ectopic expression of Oct proteins is widely used as a means to redirect and reprogram cellular identity^7–13^. Well-studied examples include reprogramming of somatic cells into induced neural stem cells (iNSCs) with Oct9 or into induced pluripotent stem cells (iPSCs) with Oct4^7,10^.

Arguably, the most studied Oct family member is Oct4, not only because of its essential physiological role in early development but also because of its unique transcriptional functions in reprogramming biology^5,14–17^. Oct4 is the only OCT protein that can induce pluripotency^18–21^, which has been a somewhat surprising revelation since OCT family members display profound sequence conservation. Notwithstanding, these observations have fueled long-standing interest in understanding exactly how OCT4 evokes pluripotency and why other Oct family members do not have this effect.

Recent studies offer exciting new insights into the biology of reprogramming both with and, interestingly, without exogenously administered Oct4. It was shown that reprogramming somatic cells to pluripotency can in fact be achieved by completely abrogating ectopic Oct4 expression^22,23^. This result indicates that reprogrammed cells acquire and establish a self-sustaining pluripotent state that is evoked by endogenous Oct4 expression. Importantly, it was shown that reprogramming without the use of exogenous Oct4 significantly improves the overall quality of the iPSCs because exogenous Oct4 can disrupt imprinted gene expression and create off-target effects^23^. Indeed, iPSCs generated without the use of exogenous Oct4 display superior developmental potential as determined by a tetraploid complementary assy. These observations, together with results from other recent studies, revealed that the induction of pluripotency can be achieved as long as endogenous Oct4 can be activated, either directly or indirectly^24–30^. Essentially, it has become clear that, while endogenous Oct4 expression is necessary, exogenous Oct4 is sufficient but not necessary for reprogramming somatic cells.

Along these lines, it was recently shown that virtually all OCT proteins harbor reprogramming competence, and they can activate the pluripotency network to different degrees under optimal conditions^31,32^; these conditions are differentially inferred by diverse donor cell epigenomes that differ not only between donor cell types but also between species. It appears that reprogramming competence not only can be significantly enhanced, for example, by chemical intervention, but can also be synthesized completely de novo via optimal combinations of chemically altered donor cell epigenomes and exogenously provided transcriptional regulators that activate the pluripotency network under defined culture conditions^31,32^. Here, we review our current understanding of how Oct proteins function in the reprogramming process and discuss the differential roles of exogenous and endogenous Oct4 in reprogramming biology. We provide an overview of factors that can replace Oct4 in murine and human reprogramming and discuss how these replacements enable the acquisition of pluripotency. We elaborate on recent findings showing how reprogramming can be achieved without administration of exogenous Oct4 and share our view on how different reprogramming competences of Oct proteins are differentially mediated in different species.

### POU proteins: an overview

The POU (Pit-Oct-Unc) protein family comprises 15 transcription factors that bind to specific target sequences^33–36^. POU proteins have diverse roles in a wide range of cellular processes^4–6^ and are categorized into six classes (POU I to POU VI) based on the sequence similarity of their DNA-binding domains (DBDs)^1,3^. Only proteins in the POU II, III and V classes, which predominantly bind to the octamer motif (consensus sequence ATGCAAAT) and its variants, are classified as octamer-binding (Oct) proteins. Proteins in other POU classes (POU I, IV and VI) display lower binding affinity for the octamer motif and thus are classified as nonoctamer-binding proteins.

The Oct protein group constitutes eight family members (Oct1, Oct2, Oct4, Oct6, Oct7, Oct8, Oct9 and Oct11), and the numbering of each protein is based on the position at which DNA probes used in electrophoretic mobility shift assays (EMSAs) are bound^2^. Oct proteins share a highly conserved bipartite DNA-binding domain (DBD), consisting of two structurally independent subdomains, a 75 amino acid N-terminal POU-specific domain (POU_S_) and a 60 amino acid C-terminal POU homeodomain (POU_H_)^1,3,4,37,38^. Each POU domain can bind a sequence of four base pairs in the major groove of the cognate DNA sequence, thereby placing each POU domain on either side of the DNA helix and effectively encircling target DNA sequences^37,38^. A linker, which varies in length and sequence between Oct proteins, is flanked by these two POU domains^4,6,37^. This linker also influences the binding specificity and conformation of Oct proteins to DNA sequences, although it does not appear to physically interact with the DNA sequence itself^39,40^. Posttranslational modifications of POU domains, including ubiquitylation, glycosylation, SUMOylation, phosphorylation and oxidation, also influence the binding ability of Oct proteins to cognate DNA sequences^41–45^. Oct proteins form homodimers or heterodimers with other partner proteins on specific target sequences^46–49^, adding an additional layer of target gene control. Oct proteins harbor two transactivation domains (TADs) that are located on each side of the DBD^5^. In contrast to DBDs, TADs exhibit little sequence conservation and vary in length between Oct proteins. TADs are known to play important roles in the transcriptional stimulation of target genes by interacting with basal transcription machinery and other cofactors^50,51^.

### Biological functions of Oct proteins in development

Oct proteins of the POU II class include Oct1 (also known as Otf-1 and Pou2f1), Oct2 (also known as Pou2f2) and Oct11 (also known as Skn-1 and Pou2f3)^4–6^. Although they belong to the same class, their expression patterns and functions are grossly different during embryonic development. Oct1 is ubiquitously expressed and can be detected in almost all cell types^52,53^, whereas the expression of Oct2 and Oct11 is largely restricted to specific cell types. Oct2 is highly expressed in B lymphocytes and plays an important role in normal germinal center reactions^54–56^. Oct11 is highly expressed in skin epithelial cells and taste cells and is critical for epidermal differentiation and the composition of taste receptor cells^57–59^.

Oct4 belongs to the POU V class^4–6^. It is the best-characterized OCT member because of its profound biological importance in early embryonic development, germ cell maintenance, stem cell pluripotency and cellular reprogramming^5,14–17^. Oct4 is highly expressed in totipotent and pluripotent cells, including oocytes, early cleavage-stage embryos, inner cell mass (ICM) of blastocysts, epiblasts, embryonic stem cells (ESCs) and germ cells^60–62^. Oct4 expression is regulated by two cis-enhancer elements (distal and proximal enhancers) that are located 2 kb upstream of its transcriptional start site^63,64^. Interestingly, the activity of these enhancers differs between cell types. The proximal enhancer (PE) is active in epiblasts of postimplantation embryos and epiblast stem cells (EpiSCs), whereas the distal enhancer (DE) is active in the ICM, ESCs and germ cells^64–66^. Reducing the expression levels of Oct4 in ESCs triggers their differentiation towards primitive endodermal, mesodermal and trophectodermal cell lineages^67,68^. Oct4-deficient embryos are viable to the blastocyst stage, but the ICM cannot be formed in these embryos^69^. These observations, together with the discovery of iPSC technology^10,70^, have underscored the functional importance of Oct4 for both the maintenance and establishment of pluripotency.

POU III factors (Oct6, Oct7, Oct8 and Oct9) are prominently expressed in cells and tissues of the central nervous system (CNS)^1,71,72^. They play critical roles in neurogenesis and gliogenesis^73–77^. Interestingly, the deletion of any one of these genes in mice does not lead to severe phenotypes^74,75,78^. Distinct phenotypes are only observed when two or three of POU III factors are simultaneously deleted, indicating their functional redundancy. The DBDs in POU III factors are highly conserved, and consequently, these factors display similar DNA-binding characteristics and DNA-dependent dimerization features^18,20,79,80^, providing a molecular explanation for their functional redundancy. Along this line, overexpression of any POU III factor can convert fibroblasts into neural lineage cells^7–9,11–13^. Thus, similar to Oct4, POU III factors are not only essential regulators of normal development but can also convert cellular phenotypes upon ectopic expression.

Oct6 (also known as SCIP, Tst-1 and Pou3f1) is expressed in Schwann cells, oligodendrocyte progenitor cells (OPCs) and keratinocytes^71,73–76,78,81^. Furthermore, Oct6 play an important role in the specification and differentiation of neuroectodermal lineages^82–85^. It was shown that Oct6 is expressed in ESCs^72,86,87^. However, we and others have recently failed to detect Oct6 expression in the ICM, iPSCs or ESCs^32,83,88–91^. Instead, its expression was detected in epiblasts of E5.5–E6.5 postimplantation embryos and EpiSCs^32,83,88–91^. Epiblasts and EpiSCs represent a more developmentally differentiated state compared to the ICM, iPSCs or ESCs and display gene expression characteristics of gastrula-stage ectoderm^66,92,93^. To date, knockout studies of Oct6 in epiblasts and EpiSCs have not been performed. Therefore, the specific role and functionality of Oct6 in epiblast formation and maintenance of the primed state of pluripotency remain incompletely understood. However, one can speculate that its role in embryonic development is not as critical as that of Oct4 because homozygous Oct6-knockout mice are born, although they die soon after birth because of respiratory defects^73,94^.

### Reprogramming of somatic cells to pluripotency

Yamanaka and Takahashi made a landmark discovery showing that ectopic expression of Oct4, Sox2, Klf4 and c-Myc can reprogram somatic cells into iPSCs^10,70^. These four reprogramming factors work interdependently to inactivate features of somatic cell identity and concurrently to activate properties of pluripotent stem cells^95–98^. During cell fate transition, cells undergo dramatic changes at the morphological, transcriptional and epigenetic levels. Notably, only a small proportion of these cells pass through all of these changes and eventually became bona fide iPSCs. Therefore, the reprogramming process is stochastic and inefficient^99^.

Various gain- and loss-of-function screenings have led to the discovery of specific genes and molecular pathways that inhibit or enhance the reprogramming process^19,26,31,100–112^. For example, specific epigenetic modifications, including DNA methylation, H3K9 methylation and H3K79 methylation marks, and/or the related enzymes (e.g., DNMTs, HDACs, LSD1, and DOT1L) can act as barriers to the reprogramming process. Therefore, forced elimination of these epigenetic barriers either through genetic inactivation or chemical inhibition can enhance or improve the reprogramming process^31,100,102,103,106–109,112^. In contrast, forced expression of chromatin remodelers, such as Dppa2 and Dppa4, can reset the epigenome of somatic cells to a pluripotent configuration that enhances both reprogramming efficiency and kinetics^113^. In addition, other transcription factors, including Esrrb, Glis1, Nr5a2, Prdm14, Rarg, Sall4, Tbx3, Foxa2, Foxf1, Foxh1, Lhx1 and T, can each significantly enhance reprogramming when overexpressed together with Oct4, Sox2, Kfl4 and c-Myc^19,26,101,104,105,110,111,114^. Other studies have shown that altering culture conditions and/or using different donor cell types can result in completely different reprogramming outcomes^115–118^. For example, adding specific nutrients (e.g., vitamin C) to the culture medium or inducing hypoxic cell culture conditions can greatly enhance reprogramming efficiency^116,118^. Furthermore, significantly higher reprogramming efficiency can also be achieved with donor cells that contain inherent phenotypic plasticity and/or high replication potential^115,117^.

Despite the significant advances that have been made to improve and understand the reprogramming process with Oct proteins, critical questions have remained unanswered, e.g., Is exogenous Oct4 expression truly essential for inducing pluripotency? More importantly, how are reprogramming competences of Oct proteins actually mediated and what are the structural bases that define them? Finally, how or why do reprogramming competences of Oct proteins differ, particularly among species?

### Exogenous Oct4, but not endogenous Oct4, is dispensable for reprogramming

For many years and for several reasons, it has been widely assumed that exogenously administered Oct4 is the most important and, in fact, indispensable factor for the reprogramming process. First, while Sox2, Klf4 and c-Myc can each be replaced by their respective family members, Oct4 cannot be replaced by any of its paralogs^18–21^. Second, and again in contrast to Sox2, Klf4 or c-Myc, Oct4 alone can elicit reprogramming when specific pathways or molecules are inhibited by compounds (e.g., VPA, a HDAC inhibitor, or CHIR-99021, a GSK-3 inhibitor) or when a specific cell type is used as a donor cell (e.g., NSCs)^119–123^. Finally, perturbation of key Oct4 functions by deletion of structural components or by introduction of inactivating point mutations completely abolishes the reprogramming process^18,20,124,125^.

**Fig. 1:**
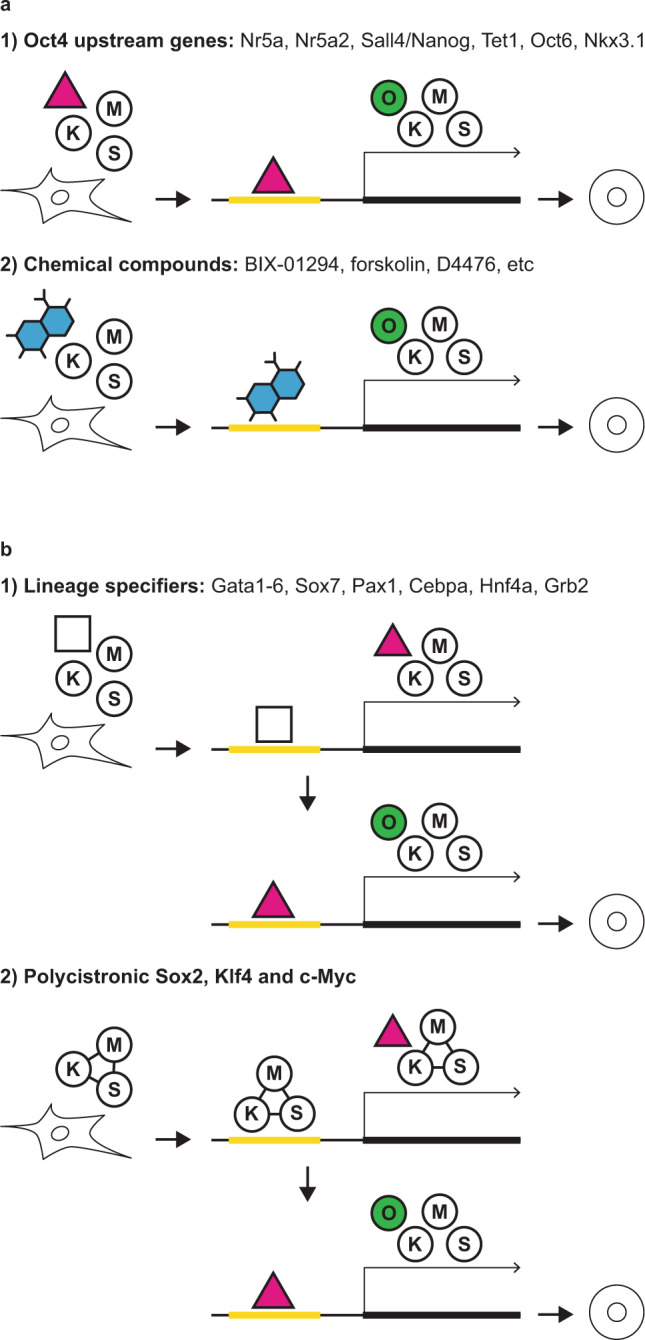
iPSCs can be generated by direct or indirect activation of endogenous Oct4. **a** Direct activation of endogenous Oct4 by Oct4 upstream genes or chemical compounds. **b** Indirect activation of endogenous Oct4 by lineage specifiers or polycistronic expression of Sox2, Klf4, and c-Myc.

However, the view that exogenous Oct4 is indispensable for reprogramming has recently changed. Undoubtedly, including Oct4 in reprogramming cocktails appears to be the most efficient means to generate iPSCs. However, recent studies have revealed that exogenous Oct4 can be entirely omitted from reprogramming cocktails or can be replaced by its family members, different transcription factors or small molecules^22,24–27,29–32,126–129^. For example, Nr5a1 (also known as Sf-1), Nr5a2 (also known as Lrh1) or Sall4/Nanog can each functionally replace Oct4 and elicit reprogramming together with Sox2, Klf4 and c-Myc^24,26^. Notably, these genes lie genetically upstream of Oct4, such that their ectopic expression can directly activate endogenous Oct4 expression, resulting in iPSC generation^24,130–132^ (Fig. 1a). The DNA demethylase Tet1 can also functionally replace Oct4 in reprogramming^25^. Ectopic expression of Tet1 mediates demethylation at the regulatory regions of Oct4, consequently resulting in activation of endogenous Oct4. These studies clearly demonstrate that the induction of pluripotency in conjunction with Sox2, Klf4 and c-Myc can be mediated without exogenous Oct4 by the use of alternative factors that are capable of directly activating endogenous Oct4 expression.

Interestingly, genes that do not directly regulate the endogenous Oct4 locus can also elicit reprogramming in conjunction with Sox2, Klf4 and c-Myc (Fig. 1b). These genes include Gata1–6, Sox7, Pax1, Cebpa, Hnf4a and Grb2, most of which, in normal physiological processes, do not lie genetically upstream of Oct4 and are not expressed in ESCs or iPSCs. Most of these factors normally function as lineage-specific transcription factors and play important roles in cell fate determination of mesodermal and endodermal lineages during embryonic development^29,30^. A study aiming to decipher the molecular mechanisms of Gata factor-based reprogramming revealed that ectopic expression of Gata factors can activate Sall4, which in turn activates endogenous Oct4, consequently resulting in iPSC generation without exogenously administered Oct4^30^. Based on these observations, it has been speculated that activating endogenous Oct4 expression indirectly may be a common way to elicit reprogramming by all other lineage-specific transcription factors. However, Nkx3.1 and Oct6, which are also lineage-specific transcription factors, do not seem to follow this mode of action. Indeed, we and others have recently revealed that Nkx3.1 and Oct6 can directly bind to regulatory regions of Oct4 to regulate its expression^32,126^. Thus, these observations clearly suggest that the indirect mode of action might not apply to the reprogramming function of all other lineage-specific transcription factors.

In addition to ectopic expression of genetic factors, specific chemicals can also functionally replace exogenous Oct4 and induce pluripotency in conjunction with Sox2, Klf4 and c-Myc^27,28^. For instance, NSCs transduced with Sox2, Klf4 and c-Myc and subsequently cultured in the presence of BIX-01294, an inhibitor of the histone methyltransferase G9a, give rise to iPSC colonies^28^ (Fig. 1a). G9a is critical for de novo DNA methylation at regulatory regions of Oct4^133,134^. Thus, BIX-01294 treatment results in demethylation at regulatory regions of Oct4, leading to activation of endogenous Oct4 expression^28^. BIX-01294-mediated reprogramming does not seem to be effective with mouse embryonic fibroblasts (MEFs), indicating that inhibition of different molecular pathways is required for eliciting reprogramming of different types of donor cells. In MEFs, exogenous Oct4 can be functionally replaced by various other small molecules, including forskolin (a cAMP agonist), 2-methyl-5-hydroxytryptamine (a 5-HT3 agonist) or D4476 (a casein kinase 1 inhibitor)^27^ (Fig. 1a). The precise molecular mechanism by which each of these chemicals induces pluripotency in conjunction with Sox2, Klf4 and c-Myc remains elusive. However, it is likely that some, or perhaps all, of these chemicals induce endogenous Oct4 expression either directly or indirectly since iPSC colonies would otherwise not be formed. With these studies, it has been established that exogenous Oct4 is dispensable for reprogramming, but endogenous Oct4 is not, which is in agreement with the fact that endogenous Oct4 is absolutely required for the establishment of pluripotency in preimplantation-stage embryos^69^.

### Reprogramming with Sox2, Klf4 and c-Myc alone produces high-quality iPSCs

We and others recently showed that iPSC generation is possible without exogenous administration of Oct4 or any of its known replacers (Oct4 upstream genes, lineage-specification transcription factors or chemicals) when Sox2, Klf4 and c-Myc are connected together by the 2 A peptide and ectopically expressed from a single polycistronic vector in MEFs^22,128^. Integrative ChIP-seq and RNA-seq analyses of cells undergoing reprogramming with Sox2, Klf4 and c-Myc alone revealed that Sox2 and Klf4 can directly bind and mediate the activation of pluripotency genes, including but not limited to Sall4 and Nanog, which in turn mediate the activation of endogenous Oct4 expression^22,128^. Interestingly, reprogramming by Sox2, Klf4 and c-Myc alone is context-dependent for various reasons. First, only polycistronic expression of Sox2, Klf4 and c-Myc can elicit reprogramming^22,128^. Monocistronic expression of Sox2, Klf4 and c-Myc does not yield iPSC colonies, indicating that stoichiometric cooperativity with these three reprogramming factors is necessary for eliciting reprogramming. Second, iPSC generation is possible by lentiviral and episomal delivery of Sox2, Klf4 and c-Myc but not by retroviral delivery due to rapid retroviral silencing that occurs in the early phase of reprogramming. Interestingly, the simultaneous expression of Sox2 and Klf4 activates retrovirus silencing machinery (e.g., Trim28, Setdb1 and Chaf1a/b), which in turn mediates retroviral silencing^128^. Finally, reprogramming with Sox2, Klf4 and c-Myc alone is species-dependent, such that reprogramming in this manner can be achieved in mouse cells but not in human cells^128^. In future studies, elucidating exactly why or how the function of these three factors differs between species will provide important mechanistic insights that will further help to define the molecular impediments to the reprogramming process.

The poor quality of iPSCs yields from reprogramming is a key limitation that precludes the development of iPSC-based applications in regenerative biomedicine. It has been shown that the quality of iPSCs can be directly influenced by the stoichiometric expression of reprogramming factors (e.g., Oct4^high^/Klf4^high^/Sox2^low^/c-Myc^low^), supplements of cell culture media (e.g., vitamin C) and/or the choice of reprogramming factors (e.g., Sall4/Nanog/Esrrb/Lin28a or Oct4/Sox2/Klf4)^135–137^. Although reprogramming with c-Myc together with Oct, Sox2 and Klf4 provides larger yields of iPSCs, c-Myc exerts a detrimental effect on imprinted loci, resulting in significantly reduced developmental potential^135^. Intriguingly, we recently discovered that the quality of iPSCs is dramatically reduced by exogenously expressed Oct4^128^. In fact, iPSC lines generated by polycistronic expression of Sox2, Klf4 and c-Myc without Oct4 produced all-iPSC mice much more efficiently than iPSC lines generated by conventional polycistronic expression of the four factors. By comparing transcriptomes and epigenomes between a series of iPSC lines generated with the two different reprogramming cocktails, i.e., with and without exogenous Oct4, we came to the decisive conclusion that ectopic Oct4 expression reduces iPSC quality by anomalously activating off-target genes unrelated to pluripotency and disrupting imprinted gene expression^128^. Considering the immense usage of iPSCs in various biomedical applications, investigations into whether iPSC lines generated without exogenous Oct4 by alternative factors are of good quality are warranted.

It has been shown that obtaining high-quality iPSCs is strongly correlated with lower reprogramming efficiency^135,136^. Consistently, reprogramming without Oct4 but with Sox2, Klf4 and c-Myc produces fewer iPSC colonies with slower reprogramming kinetics, but the majority of the iPSC lines derived from these colonies are of superior quality, as assessed by molecular profiling and their contribution to tetraploid complementation^128^. Thus, the theme that “less is more” appears to hold true for reprogramming biology. Reprogramming systems that yield iPSCs with high efficiency and fast kinetics come with the price of generating a high proportion (too high) of low-quality iPSCs.

### Transactivation domains and donor-cell epigenomes are tightly linked to reprogramming competences

Despite the iPSC quality drawbacks that can occur by reprogramming with exogenous Oct4, it is by far the strongest pluripotency inducer. The observation that Oct4 cannot be functionally replaced by other Oct family members in murine reprogramming^18,20,21,31^, although Oct proteins share profound sequence conservation, has fueled interest in understanding what makes Oct4 uniquely competent in inducing pluripotency. We and others have compared the structures and biochemical properties of Oct proteins to better understand the molecular basis of the reprogramming processes. Interestingly, despite their high similarities in protein sequences, the DNA-binding profiles of Oct proteins and their DNA-dependent dimerization with Sox2 are remarkably different^18,20,80^. Specifically, Oct4 preferentially forms heterodimers with Sox2 through the canonical SoxOct motif (CATTGTTATGCAAAT), which is highly enriched in enhancers of pluripotency genes, including Nanog, Fgf4, Utf1 and Pou5f1^18,46,47,80^. In contrast, other Oct proteins, such as Oct6 and Oct7, preferentially form homodimers through the more palindromic octamer recognition element (MORE; ATGCATATGCAT) motif, which is predominantly found in the regulatory regions of neural genes, including Neurog1, Olig1 and Ascl1^18,47–49^. The formation of Oct4-Sox2 heterodimers through SoxOct motifs appears to be necessary for inducing pluripotency because mutations that disrupt Oct4-Sox2 heterodimer formation abolish reprogramming competence^18,138,139^. Oct6 and Oct7 can also bind to the SoxOct motif in EMSAs, albeit with low efficiency^18,80^. However, ChIP assays with ESCs, neural precursor cells (NPCs) and cells undergoing reprogramming failed to detect Oct6 and Oct7 binding to the SoxOct motif^13,20,32,77,80,140^. The discrepancy of these results might be due to the different experimental settings and sensitivities of these two different assays. Notwithstanding, these studies clearly indicate that Oct proteins display differential DNA-dependent dimerization properties and DNA-binding profiles, providing an explanation for why they display different reprogramming competences and, more specifically, why Oct4 cannot be functionally replaced by its family members in murine reprogramming.

To support this idea, through structural modeling, we found that methionine at position 151 is critical for the homodimerization of Oct proteins through the MORE motif^18^. This amino acid is contained in POU III factors (e.g., Oct6) but not in Oct4. Therefore, amino acid substitution of methionine 151 with serine (Oct4^S151M^) increases its ability to form homodimers through the MORE motif^18^. In contrast, amino acid substitution of methionine to serine in residue 151 of Oct6 (Oct6^M151S^) reduced its ability to form homodimers through the MORE motif^18^. Although the binding propensities of these two mutants to the MORE motif were significantly altered, their respective binding propensity for the SoxOct motif was not substantially changed compared to that of their wild-type counterparts^18^. As such, Oct4^S151M^ still produced iPSC colonies similar to wild-type Oct4 and Oct6^M151S^ was unable elicit reprogramming similar to wild-type Oct6^18^. These findings indicate the binding preference of Oct proteins for MORE is not the key to their reprogramming competences. Previously, it was shown that two amino acids at residues 7 and 22 in the POU-specific domain of Oct4 are essential to form an Oct4-Sox2-DNA ternary structure^141^. Interestingly, changing these amino acids by substituting aspartic acid with lysine at residue 7 and lysine with threonine at residue 22 in Oct6^M151S^ (OCT6 ^M151S/D7K,K22T^) enables this engineered Oct6 mutant to bind to the SoxOct motif. Consequently, it is capable of producing iPSC colonies^18^. However, the reprogramming efficiency of this mutant was extremely low. Thus, other structural features in addition DNA-dependent dimerization are required to mediate Oct6-mediated reprogramming efficiently in murine cells.

In mouse ESCs, Oct4 mutants in which both N-TAD and C-TAD are deleted show severe phenotypes and cannot maintain a pluripotent state^142^. Interestingly, only one TAD in Oct4 is needed to maintain the functionality of Oct4, since mouse ESCs in which either the N-TAD or C-TAD of Oct4 is deleted maintain pluripotency^142^. However, when the reprogramming competences of Oct6-Oct4 domain-swapped chimeras were tested, the chimeric proteins (O466 and O664) in which either N- or C-TADs of Oct4 was introduced into the corresponding site of Oct6 did not acquire reprogramming activity^32^. However, when both N- and C-TADs of Oct4 were introduced into corresponding sites of Oct6, the chimeric protein (O464) induced the formation of iPSC colonies^32^. Overall, these observations reveal interconnected functional features of TAD and DBD, which together are crucial for mouse cell reprogramming and pluripotency maintenance: differential DNA-dependent binding propensities through the DBD and functional features of the TAD that are yet to be discovered.

### OCT6 is a pluripotency inducer and acts as a pioneer transcription factor in human reprogramming

Oct4 has been used for iPSC generation from cells obtained from a number of different species, including mice, humans, rats, monkeys and gorillas^10,70,143–145^, underscoring the universality and conserved fundamental function of Oct4 for inducing pluripotency. Notably, most studies addressing the possibility of replacing Oct4 for reprogramming have been performed with mouse cells^24–26,29,30^. However, because of its ever more increasing significance for biomedicine, there is great interest in determining whether Oct4 replacers can function similarly in cells from other species, especially humans. Until recently, Nxk3.1 was the only known factor that can induce pluripotency in addition to Oct4 in both human and mouse cells^126^. Nr5a1, Nr5a2, Tet1, Sall4, Nanog and Gata3 can all replace Oct4 in reprogramming murine cells to pluripotency. However, these paralogs fail to produce iPSC colonies in conjunction with Sox2, Klf4 and c-Myc in human cells^32^, clearly indicating their mouse-specific reprogramming competence. A potential explanation for these divergence outcomes might involve the differences in the chromatin structures and epigenomes in human and mouse cells. This is because the accessibility of reprogramming factors to target sites differ between human and mouse cells^146–149^. Thus, the reprogramming competences of these Oct4 replacers are likely differentially regulated depending on the epigenetic state of the donor cells. In agreement with this concept, we recently showed that creating an epigenetically permissive environment for human donor cells through a combinational treatment of compounds that affect chromatin modifications, including DNA methylation and histone methylation, enabled the generation of iPSC colonies upon administration of Nr5a1, Nr5a2, Tet1, Sall4, Nanog and Gata3^31^. Together, these findings triggered our hypothesis that other factors which can replace Oct4 specifically in human reprogramming might exist.

By executing a functional screening of 100 tested candidate genes, we surprisingly found that Oct6 can induce pluripotency in conjunction with Sox2, Klf4 and c-Myc in human cells^32^. This observation was especially intriguing because Oct6 cannot generate iPSC colonies from mouse cells^18,20,21^. Notably, human and mouse Oct6 proteins share 98.89% identity, and both proteins can elicit reprogramming of human cells but not of mouse cells^32^. Therefore, its species-dependent reprogramming competence is not mediated by sequence variations of orthologous Oct6 proteins but by the differences in mouse and human cell epigenomes. Indeed, if the levels of specific epigenetic modifications, including H3K27 methylation, H3K79 methylation, H3K4 methylation and H3K9 methylation, are modulated, ectopic Oct6 expression enables iPSC generation from mouse cells^31^.

Although Oct4 and Oct6 are functionally interchangeable for inducing human pluripotency, OCT6-mediated reprogramming is significantly slower and less efficient than that mediated by OCT4^32^. Comparative RNA-seq analysis of TRA-1–60^+^/CD31^−^ cells collected over the time course of reprogramming with Oct4 and Oct6 revealed that the mesenchymal-to-epithelial transition (MET) was not different. This finding supports the argument that Oct proteins do not affect or delay the MET process during reprogramming. However, the activation of pluripotency genes is remarkably delayed in Oct6-mediated reprogramming^32^. Integrative ChIP-seq and RNA-seq analyses revealed that the delayed activation of pluripotency genes in Oct6-based reprogramming is largely due to the delayed binding of Oct6 to regulatory regions of corresponding genes^32^. Consequently, Oct6-mediated reprogramming is relatively inefficient and slow. Importantly, Oct6 can directly bind to the Oct4 locus, underscoring the concept that endogenous Oct4 expression is essential to the reprogramming process. Therefore, Oct6 lies genetically upstream of Oct4, at least in humans. In mice, Oct4 and Oct6 are coexpressed in EpiSCs and epiblasts^32,83,88–91^. Therefore, in future studies, it will be important to investigate whether and how Oct4 is regulated by Oct6, or vice versa, in these specific cell types to better understand the species-specific impediments of reprogramming with these factors.

Oct4 fulfills the criteria of being a pioneering transcription factor, such that it can engage nucleosomal DNA and thereby initiate regulation of its target genes that promote a change in cell fate^148–150^. To date, exactly how this pioneering activity is achieved remains poorly understood. We recently showed that the pioneering activity of Oct4 depends on functional synergism between Oct4 C-TAD and Oct4 DBD^32^. The absence of Oct4 C-TAD significantly abolishes the ability of Oct4 to bind pluripotency gene enhancers, and more importantly, its function is only possible when it is connected to the Oct4 DBD. In fact, introducing Oct4 C-TAD to corresponding sites of Oct6 does not change the binding of Oct6 to pluripotency gene enhancers and its reprogramming competence^32^. Intriguingly, in contrast to Oct4, Oct6 displays its pioneering synergistic activity with its DBD through the N-TAD. Interestingly, the Oct4 C-TAD can be functionally replaced by the Oct6 N-TAD but not the Oct6 C-TAD^32^. Similarly, when the N-TAD of Oct6 is connected with Oct4 DBD, the binding ability of these chimeras to enhancers of pluripotency genes dramatically increases. It has been shown that the N-TAD of Gata3 is critical for Gata3-mediated chromatin remodeling and its pioneering activity^151^. The C-TAD of Ebf is also required for its pioneering activity and B cell reprogramming^152^. Furthermore, the C-TAD of Hnf3 (also known as FoxA) is critical for its pioneering function^153^. Overall, these observations support our conclusion that the C-TAD of Oct4 and the N-TAD of Oct6 are critical for their pioneering activities and reprogramming competences.

## Concluding remarks

Reprogramming somatic cells to pluripotency is, in fact, a complicated process, and this process does not rely on a single molecular pathway. Our recent findings together with those of other studies clearly suggest that molecular pathways and roadmaps to reach pluripotency can be diverse, depending on the epigenetic state of the donor cells and the exogenously provided transcription factors. Therefore, modulating epigenomes by chemical intervention and introducing different combinations of transcription factors in different donor cell types and also in different species might further enhance our understanding of reprogramming mechanisms.
